# Mechanical, Morphological and Antimicrobial Properties of Mango-Corn and Mango-Potato Seed Starch Polymers

**DOI:** 10.3390/polym18172076

**Published:** 2026-08-27

**Authors:** Moises Gallozzo-Cardenas, Renny Nazario-Naveda, Luis Angelats-Silva

**Affiliations:** 1 Dirección de Instituto y Centros de Investigación, Universidad César Vallejo, Trujillo 13001, Peru; 2 Dirección de Investigación, Universidad César Vallejo, Trujillo 13011, Peru; 3 Laboratorio de Investigación Multidisciplinario, Universidad Privada Antenor Orrego, Trujillo 13008, Peru

**Keywords:** antimicrobial activity, biopolymers, biodegradation, corn starch, mango starch, potato starch

## Abstract

The objective of this research was to evaluate the mechanical, morphological and antimicrobial properties of mango starch films with corn starch and potato starch substitution. The mango starch substitution percentages were 25%, 50% and 75% for both corn and potato starch. The films obtained were characterized by FTIR spectrophotometry and scanning electron microscopy. It was corroborated that mango starch improves its properties with the incorporation of corn and potato starch, showing a significant increase in the uniformity of the sample and in its elasticity, obtaining a mechanical stress of 23.94 MPa. The films showed a fast biodegradability process and good antimicrobial activity, which was characterized by halo inhibition technique. All the results demonstrate a great improvement of the mango starch films highlighting a good antimicrobial activity, which potentiates them as a sustainable alternative to be applied in biomedicine. The inclusion of pure commercial starches (100M and 100C) confirmed the dose-dependent evolution of properties, establishing a complete compositional baseline.

## 1. Introduction

The massive accumulation of petroleum-derived plastics represents a critical ecological challenge for global biodiversity [1,2]. It is estimated that more than 300 million tons of plastic waste are generated annually, a significant proportion of which comes from disposable packaging that pollutes terrestrial and marine ecosystems [1,3]. In 2020, nearly 86 million tons of this waste ended up in landfills, where its structural persistence can range from 10 to 600 years before degradation occurs [4]. This chemical stability promotes fragmentation into microplastics, which release harmful substances and threaten human health by entering the food chain [4,5]. The problem is severe considering that more than 90% of global plastic production between 2018 and 2022 was fossil-based, contributing substantially to greenhouse gas emissions [6]. Given the decreasing capacity of landfills and the toxicity of conventional polymers, it is imperative to investigate alternatives that harmonize modern lifestyles with environmental protection [2,4,6].

The search for sustainable solutions is vital to mitigate dependence on non-renewable resources and pollution from solid waste [7,8]. Bioplastics, defined as bio-based materials, biodegradable materials, or both, have emerged as the most viable alternative, with annual growth rates projected to exceed 200% by 2026 [4,9]. Among natural polymers, starch stands out for its abundance, low cost, and complete biodegradability in diverse environments [7,10,11]. Current methods to overcome its intrinsic limitations, such as low mechanical strength and high moisture sensitivity, include plasticization with polyols, blending with synthetic biopolymers such as polylactic acid (PLA), and reinforcement with nanomaterials or natural fibers [5,6,12]. These strategies aim to transform native starches into functional matrices through solvent casting, extrusion, and molding processes, optimizing their properties for industrial applications in active packaging and biomedical devices [5,8,9].

Several studies have laid the groundwork for the development of these materials. Gürler et al. (2021) modified potato starch through silane crosslinking and PLA bilayers, achieving an increase in tensile strength from 1.017 MPa to 10.918 MPa [3]. Ren et al. (2017) prepared corn starch–chitosan blends by melt processing, determining that an optimal chitosan concentration of 41% maximized elongation and improved water vapor barrier properties (as cited in [3]). Wahab et al. (2021) investigated cassava starch films using plasticizers such as glycerol and sucrose, concluding that the use of higher molecular weight additives results in smoother surface morphology and increased polymer stiffness [13]. Finally, Chen et al. (2018) evaluated biodegradable composites reinforced with sisal fibers, finding that oxidative esterification of starch improved the Young’s modulus by 54.3% compared to native starch [5].

The study of mechanical, morphological, and antimicrobial properties of starch-based polymers derived from mango seed starch blended with corn and potato is justified by the need to valorize agro-industrial residues within a circular economy framework. Mango starch, as a byproduct, offers a renewable source that, when combined with commercial starches, can compensate for individual structural deficiencies [4,14]. Molecular architecture, defined by the amylose/amylopectin ratio, is crucial; high amylose levels favor the formation of compact crystalline networks that enhance mechanical strength, whereas amylopectin facilitates flexibility but limits load transfer [10,11,15]. Morphology, analyzed by scanning electron microscopy (SEM) and X-ray diffraction (XRD), enables verification of mixture homogeneity and interfacial compatibility, critical factors for material integrity [10,12,13]. Furthermore, the incorporation of antimicrobial properties is essential for the development of active packaging capable of inhibiting bacterial and fungal growth, extending product shelf life, and improving food safety [4,8,14]. Assessing how these different botanical sources interact allows the development of biopolymers with enhanced thermal stability and competitive mechanical performance compared to conventional plastics [10,13].

Despite advances in binary starch blends, a knowledge gap persists regarding the specific synergistic interaction between mango seed starch and corn or potato systems in terms of morphological compatibility and antimicrobial efficacy [10].

To establish a complete compositional baseline, pure corn starch (100M) and pure potato starch (100C) films were also prepared and characterized alongside the blended formulations, enabling a comprehensive understanding of the property evolution from pure mango starch to pure commercial starches.

The primary objective is to evaluate the mechanical, morphological, and antimicrobial properties of mango starch polymers blended with corn and potato. Secondarily, the objective is to optimize their microstructure. This research is important to promote the use of agricultural residues and provide high-performance, environmentally friendly alternatives [4,14]. It also aims to establish their viability for advanced functional materials such as biomedical patches.

## 2. Materials and Methods

### 2.1. Manufacture of Films

To prepare the films, 2 g of starch extracted according to the method described by Nazario et al. [16] was carefully weighed and dispersed in 20 mL of distilled water, previously measured with a graduated pipette. The suspension was subjected to continuous magnetic stirring for approximately 10 min, until a completely homogeneous, lump-free mixture was obtained.

Subsequently, 0.8 mL of glycerol (J.T. Baker, Phillipsburg, NJ, USA, 99%, CAS: 56-81-5), used as a plasticizer and gelatinizing agent, and 1 mL of glacial acetic acid (Merck, Darmstadt, Germany, 99%, CAS: 64-19-7) were gradually added, maintaining constant stirring to ensure uniform dispersion of the additives in the starch matrix. The resulting mixture was heated in a water bath at 60 °C to initiate starch gelatinization. During heating, a 5% NaOH solution (Merck, 98%, CAS: 1310-73-2) was slowly introduced, carefully controlling the volume with a micropipette, until a pH of 7 was reached. This adjustment was crucial to ensure the stability of the mixture and promote the formation of a more consistent polymer network.

Once gelatinization began, the viscous solution was poured evenly into standard Petri dishes, ensuring complete coverage and uniform film thickness. The dishes were placed in an oven at 50 °C for 48 h, allowing sufficient time for plasticization and controlled drying, preventing the formation of bubbles or surface irregularities.

At the end of the process, the films were carefully removed from the Petri dishes with plastic tweezers to avoid mechanical damage. The samples were then cut into regular sections with a sterile scalpel, ensuring well-defined edges and reproducible dimensions for subsequent physicochemical and mechanical characterization. The starch content used in each trial was determined according to the experimental conditions specified in Table 1, thus ensuring reproducibility and comparability between the different samples.

### 2.2. Characterization of the Films

Fourier Transform Infrared Spectroscopy–Attenuated Total Reflectance (FTIR–ATR):

Functional group identification in the films was performed using FTIR–ATR with a Thermo Scientific IS50 spectrometer (Thermo Fisher Scientific, Madison, WI, USA). Each sample was placed in the analysis cell and scanned 20 consecutive times to enhance the signal-to-noise ratio. The spectral range was set from 4000 cm^−1^ to 400 cm^−1^, with a resolution of 4 cm^−1^, enabling the acquisition of detailed spectra of molecular vibrations characteristic of the bonds present in the polymeric matrix. Spectra were processed using the instrument’s software to identify representative peaks corresponding to hydroxyl groups, carbonyl groups, and C–O bonds, all relevant to the characterization of biopolymers.

Scanning Electron Microscopy (SEM):

Morphological evaluation was conducted on 1 × 1 cm^2^ samples, which were previously coated with a thin layer of carbon using a vacuum coater to improve surface conductivity and prevent charge accumulation during analysis. Images were obtained with a Tescan Vega 3 LMU microscope (TESCAN, Brno, Czech Republic), operated at an accelerating voltage of 5 kV, allowing observation of surface topography, pore distribution, and film homogeneity.

Opacity by UV-Vis Spectrophotometry:

Film opacity was determined using a Shimadzu UV-1900 spectrophotometer (Shimadzu Corporation, Tokyo, Japan), with air as the reference. Measurements were performed at a fixed wavelength of 600 nm, within the visible range. Opacity was calculated as the ratio between absorbance at 600 nm and film thickness (mm), the latter measured with a digital micrometer. This parameter provided an assessment of the relative transparency of the films and their potential application in biodegradable coatings or packaging. All measurements were performed in triplicate, and results are expressed as mean ± standard deviation (SD).

Soil Biodegradability:

Biological degradation was evaluated following the methodology described by Sachdeva et al. and other authors [17,18]. Film fragments of 2 × 2 cm^2^ were buried at a depth of 2 cm in cultivated soil under controlled moisture conditions. Every 7 days, samples were carefully retrieved, rinsed with distilled water to remove soil residues, dried at room temperature, and weighed using an analytical balance. Mass loss was recorded as an indicator of progressive biodegradation. All measurements were performed in triplicate, and results are expressed as mean ± standard deviation (SD).

Mechanical Properties:

Mechanical testing was carried out in accordance with ASTM D638. Films were cut into V-shaped dumbbell specimens with a width of 3.18 ± 0.5 mm, using a standardized die. Tensile strength and elongation were measured with a tensile testing system equipped with a Vernier Dual-Range sensor, with a precision of ±0.01 N, operating at a constant speed of 2 mm/s. Each test was repeated four times per film type, ensuring statistical reproducibility of the results. All measurements were performed in triplicate, and results are expressed as mean ± standard deviation (SD).

Antimicrobial Activity:

The antimicrobial capacity of the films was assessed using the agar well diffusion method. Fresh cultures of *E. coli* (ATCC 25922), *S. aureus* (ATCC 25923), and *C. albicans* (ATCC 10231) were prepared in Mueller–Hinton agar (for bacteria) and Sabouraud dextrose agar (for fungus), adjusted to a 0.5 McFarland standard (~1.5 × 10^8^ CFU/mL). Film fragments of 6 mm diameter were placed on the agar surface. A blank disk (without film) was used as a negative control, and disks impregnated with ampicillin (10 µg) and amphotericin B (10 µg) were used as positive controls for bacteria and fungi, respectively. Plates were incubated for 24 h at 37 °C (bacteria) and 30 °C (fungus). The inhibition zone diameters (IZD) were measured using a digital caliper. All assays were performed in triplicate, and results are expressed as mean ± standard deviation (SD). Statistical analysis was performed using one-way ANOVA followed by Tukey’s post hoc test (*p* < 0.05) using GraphPad Prism 9.0 software.

## 3. Results and Discussion

### 3.1. Fourier Transform Infrared Spectroscopy (FTIR)

**Figure 1:**
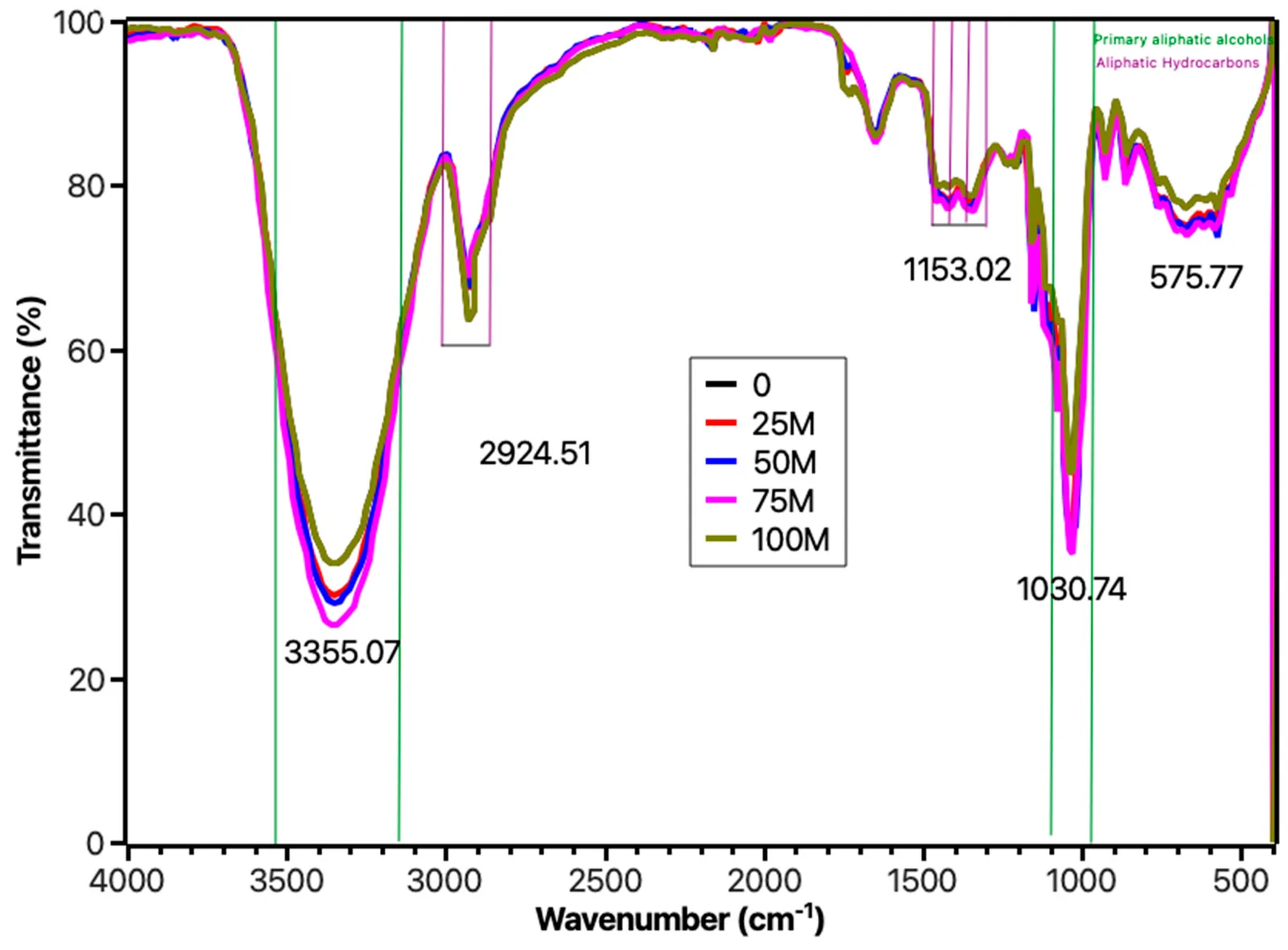
FTIR spectrum of starch films derived from mango seed and corn.

**Figure 2:**
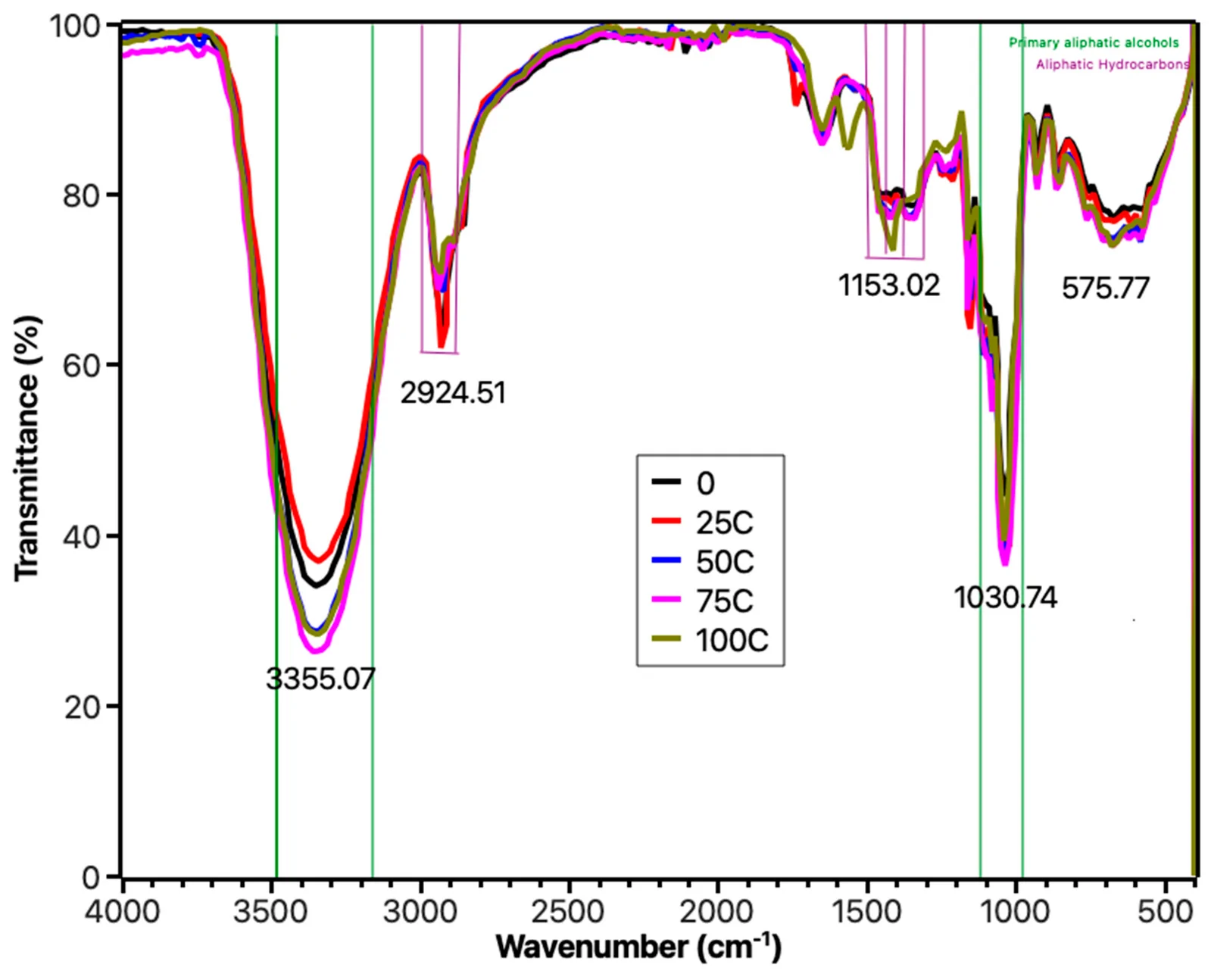
FTIR spectrum of starch films derived from potato and corn.

Figure 1 and Figure 2 show the FTIR spectra of the blended starch films. The spectra exhibit the characteristic functional groups of carbohydrate polymers, with notable variations in peak position, intensity, and bandwidth that provide molecular-level evidence of the intermolecular interactions governing blend performance [19].

O–H Stretching Region (3600–3000 cm^−1^):

The broad and intense band centered at ~3355 cm^−1^ is attributed to the stretching vibrations of hydroxyl groups (–OH), involved in inter- and intramolecular hydrogen bonding [20,21]. As the corn starch content increases (samples 25M → 75M), this band exhibits a systematic shift to lower wavenumbers (from 3360 cm^−1^ for sample 0 to 3342 cm^−1^ for sample 75M), accompanied by a progressive broadening (bandwidth increases from 342 cm^−1^ to 418 cm^−1^) [14,22]. This behavior is mechanistically significant for three reasons:

(i) Shift to lower wavenumbers: A decrease in the O–H stretching frequency indicates a strengthening of the hydrogen-bonding network. According to the Badger–Bauer rule, a shift to lower wavenumbers corresponds to shorter O···H distances and stronger hydrogen bonds, as the electron density of the O–H bond is redistributed upon hydrogen bond formation. The progressive shift from 3360 cm^−1^ (sample 0) to 3342 cm^−1^ (75M) suggests that the addition of corn starch promotes stronger inter-chain hydrogen bonds between mango and corn starch amylose chains.

(ii) Band broadening: The observed broadening is indicative of increased heterogeneity in the hydrogen-bonding environment. As the corn starch content increases, the polymer network becomes more complex, with a wider distribution of O–H bond strengths (ranging from weak to strong hydrogen bonds). This broadening is consistent with the formation of a more densely crosslinked network, where amylose chains adopt multiple conformations with varying degrees of hydrogen bonding.

(iii) Mechanical implications: Stronger hydrogen bonding reduces chain mobility, increases the glass transition temperature (Tg), and enhances the cohesive energy density of the matrix. This directly correlates with the observed 13-fold increase in tensile strength (from 1.78 MPa for sample 0 to 23.94 MPa for sample 75M).

In contrast, the potato starch blends (25C–75C) exhibit less pronounced shifts (from 3360 cm^−1^ to 3351 cm^−1^) and minimal broadening (bandwidth increases from 342 cm^−1^ to 378 cm^−1^), suggesting limited molecular miscibility between mango and potato starch polymers. This is likely due to the higher phosphate ester content and branching frequency of potato amylopectin, which introduce steric hindrance and disrupt the hydrogen-bonding network [20,21].

C–H Stretching Region (2924 cm^−1^):

The peak observed at 2924.51 cm^−1^ corresponds to the C–H stretching vibrations of sp^3^ hybridized carbons within the glucopyranose rings. The intensity of this band increases with corn starch content (from 0.82 a.u. for sample 0 to 1.15 a.u. for sample 75M), reflecting the higher hydrocarbon content of the blended matrix. The position of this band remains relatively constant (2924.51 ± 2 cm^−1^), indicating that the C–H bonds are not significantly affected by the blending process.

Fingerprint Region (1200–900 cm^−1^):

The fingerprint region provides crucial information about the amorphous/crystalline structure of the starch films. The bands at 1153.02 cm^−1^ and 1030.74 cm^−1^ are characteristic of C–O stretching vibrations:

The 1153 cm^−1^ band is associated with the asymmetric stretching of the C–O–C glycosidic linkage, which is sensitive to the conformation of the polymer backbone.

The 1030.74 cm^−1^ peak reflects the C–O stretching in C–O–H groups, which is sensitive to the amorphous/crystalline ratio of the polymer matrix [20].

A key observation is the systematic decrease in the I_1030_/I_1153_ intensity ratio with increasing corn starch content (from 1.82 for sample 0 to 1.41 for sample 75M). This decrease is mechanistically significant because:

(i) Indication of increased crystallinity: A lower I_1030_/I_1153_ ratio suggests a higher degree of molecular order, as the 1030 cm^−1^ band (associated with amorphous regions) decreases in intensity relative to the 1153 cm^−1^ band (associated with ordered structures). This is consistent with the formation of V-type amylose crystallites, where amylose chains adopt helical conformations stabilized by hydrogen bonds.

(ii) Correlation with mechanical properties: The reduction in the I_1030_/I_1153_ ratio from 1.82 to 1.41 correlates with the progressive increase in tensile strength (R^2^ = 0.92), indicating that structural ordering at the molecular level directly enhances macroscopic mechanical performance.

(iii) Correlation with opacity: The increased crystallinity reduces light scattering, as crystalline domains scatter light less intensively than amorphous regions with density fluctuations. This is consistent with the observed reduction in opacity (from 6.01 mm^−1^ for sample 0 to 3.74 mm^−1^ for sample 75M).

Low-Frequency Region (575.77 cm^−1^):

The low-frequency band at 575.77 cm^−1^ is attributed to skeletal vibrations and pyranose ring deformations. This band shows slight shifts with corn starch content (from 575.77 cm^−1^ to 578.23 cm^−1^), indicating changes in the ring conformation and torsional angles of the glycosidic linkages. These subtle changes reflect the molecular rearrangements that occur upon blending, consistent with the morphological transitions observed by SEM.

In general, the systematic analysis of peak shifts, intensity changes, and bandwidth variations reveals a clear structure–property relationship: increasing corn starch content promotes stronger hydrogen bonding (evidenced by the shift and broadening of the O–H band), higher molecular order (evidenced by the decrease in the I_1030_/I_1153_ ratio), and improved chain packing. These molecular changes collectively explain the observed improvements in mechanical performance, optical clarity, and morphological uniformity of the corn starch blends.

### 3.2. Scanning Electron Microscopy (SEM)

**Figure 3:**
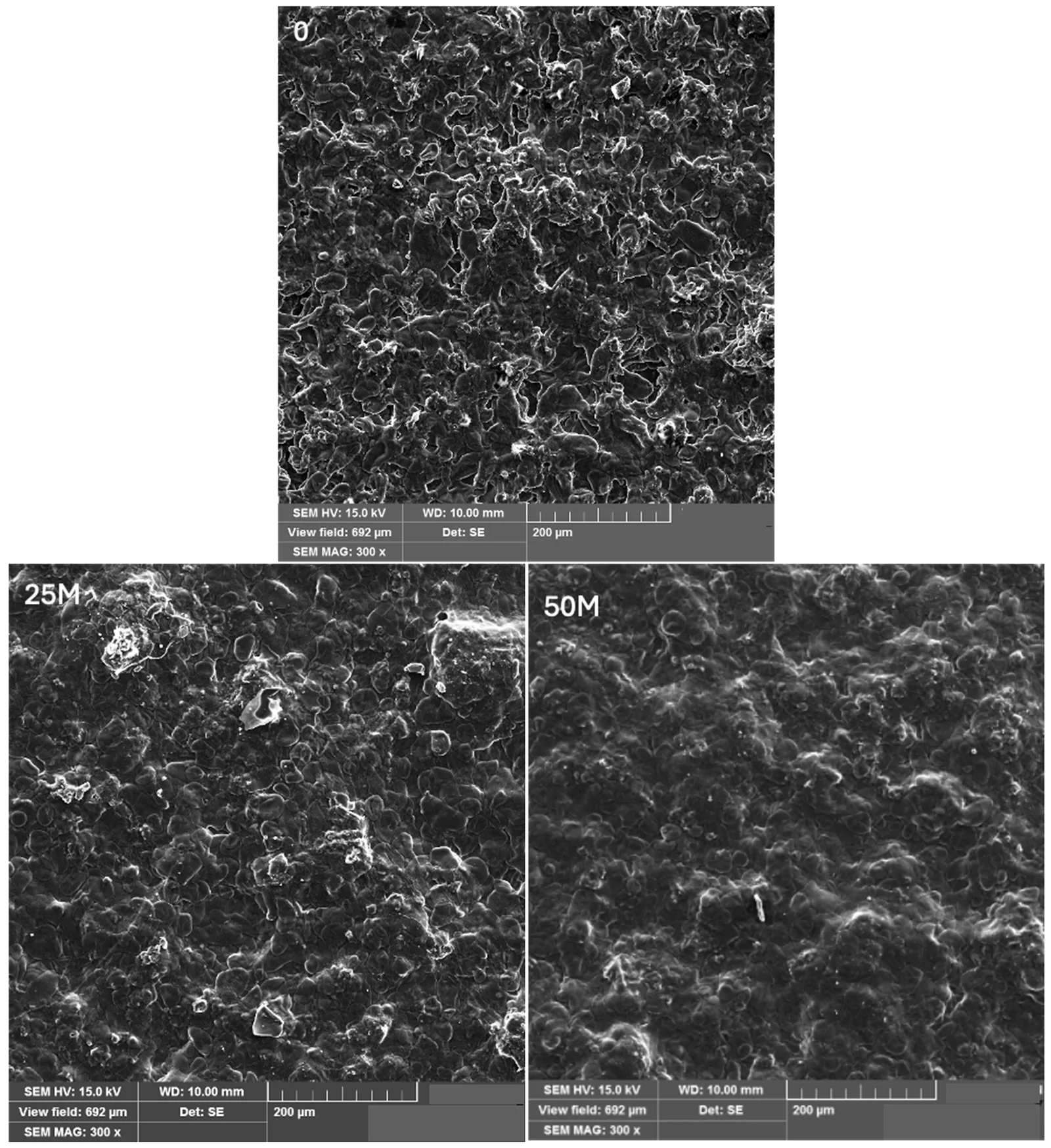

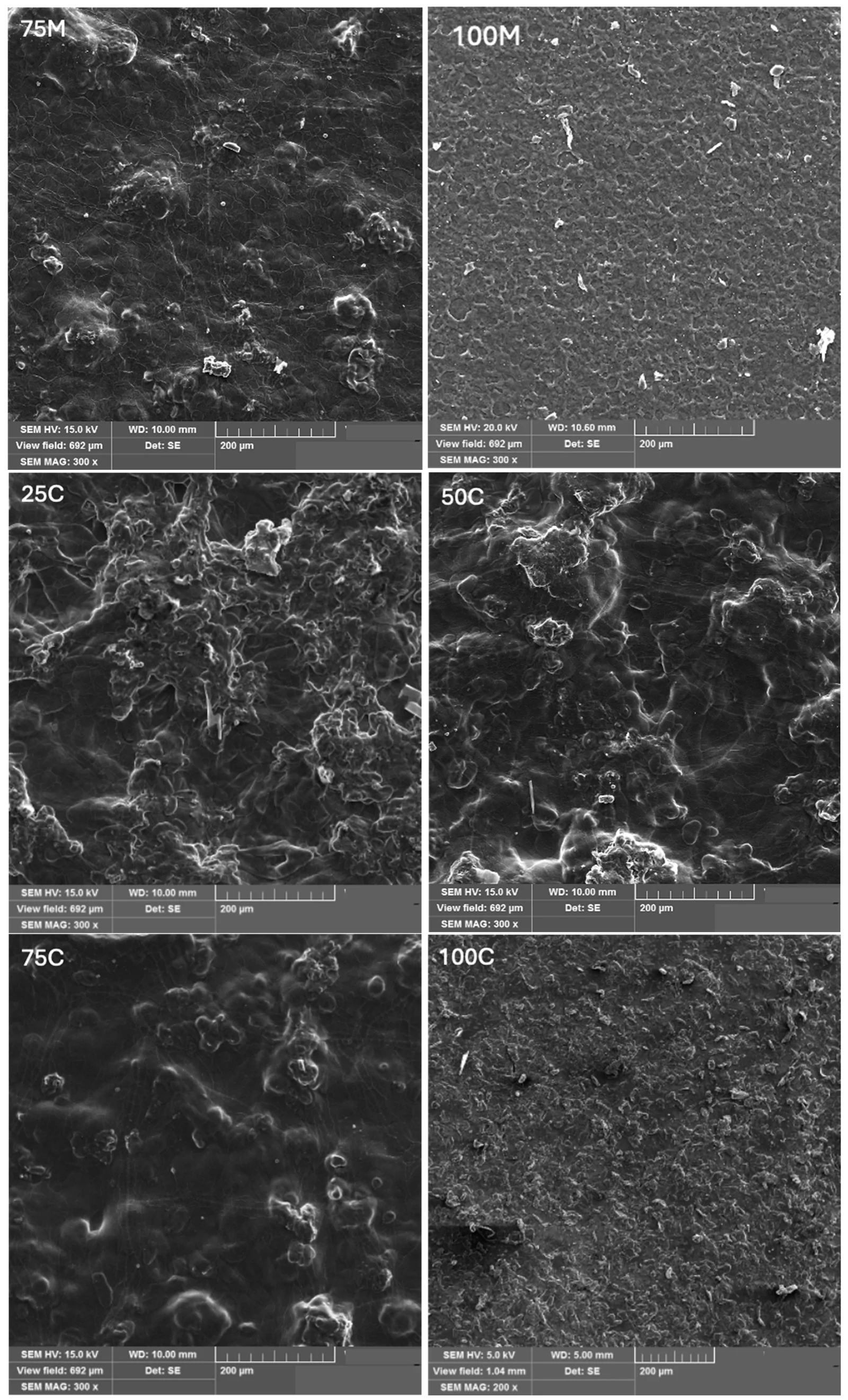
Scanning electron microscopy (SEM) images showing the morphological structure of starch-based films composed of mango seed starch blended with corn and potato starch.

Figure 3 displays the images obtained through scanning electron microscopy (SEM).

The morphological evolution observed by SEM is mechanistically linked to the gelatinization dynamics and phase compatibility of the starch blends. The rough, porous, and fibrous structure of pure mango starch (sample 0) arises from the retrogradation of amylose-rich domains during drying, which promotes micro-phase separation between amylose and amylopectin fractions. This heterogenous morphology creates stress concentration points that initiate crack propagation under tensile load, explaining the low mechanical strength (1.78 MPa).

Upon incorporation of corn starch (samples 25M–75M), a progressive transition from a fibrillar to a continuous, dense, and homogeneous matrix is observed. This is mechanistically attributed to:

(i) Molecular compatibility: The similar amylose/amylopectin ratio and molecular weight distribution between mango and corn starches facilitate co-crystallization during gelation, forming a bicontinuous interpenetrating network.

(ii) Plasticizer redistribution: Glycerol, used as plasticizer, is more uniformly distributed in the blend, reducing the plasticizer-rich domains that would otherwise create defects.

(iii) Reduced retrogradation: The presence of corn starch modulates the recrystallization kinetics, preventing the formation of large spherulites and promoting a fine, microcrystalline structure.

The improved morphological uniformity directly translates into enhanced mechanical performance: the reduction in surface defects minimizes stress concentration, increases the effective load-bearing cross-section, and delays crack initiation. This is quantitatively reflected in the 13-fold increase in tensile strength for the 75M sample.

In contrast, potato starch blends (25C–75C) maintain a partially granular structure, indicating incomplete gelatinization or poor interfacial adhesion. This is likely due to the higher gelatinization temperature and greater phosphate content of potato starch, which interfere with the homogeneous swelling and disruption of starch granules during processing. This morphological change may be associated with the emergence of the peak around 576 cm^−1^ in the FTIR spectra shown in Figure 1 and Figure 2 [5]. Although improvements in morphological characteristics are evident in both starch combinations, the incorporation of corn starch leads to greater film uniformity. The transition from a rough, porous structure (sample 0) to a dense, continuous matrix (75M) can be mechanistically explained by three concurrent factors: (i) molecular compatibility between mango and corn starches, which promotes co-crystallization during gelation and forms an interpenetrating network; (ii) uniform redistribution of glycerol, which reduces the formation of plasticizer-rich domains that act as defects; and (iii) modulation of retrogradation kinetics by corn starch, which prevents the formation of large spherulites and promotes a fine, microcrystalline structure. This morphological optimization reduces stress concentration points, delays crack initiation, and quantitatively explains the 13-fold increase in tensile strength. This enhanced uniformity is reflected in the opacity measurements presented in Table 2.

### 3.3. Opacity

**Figure 4:**
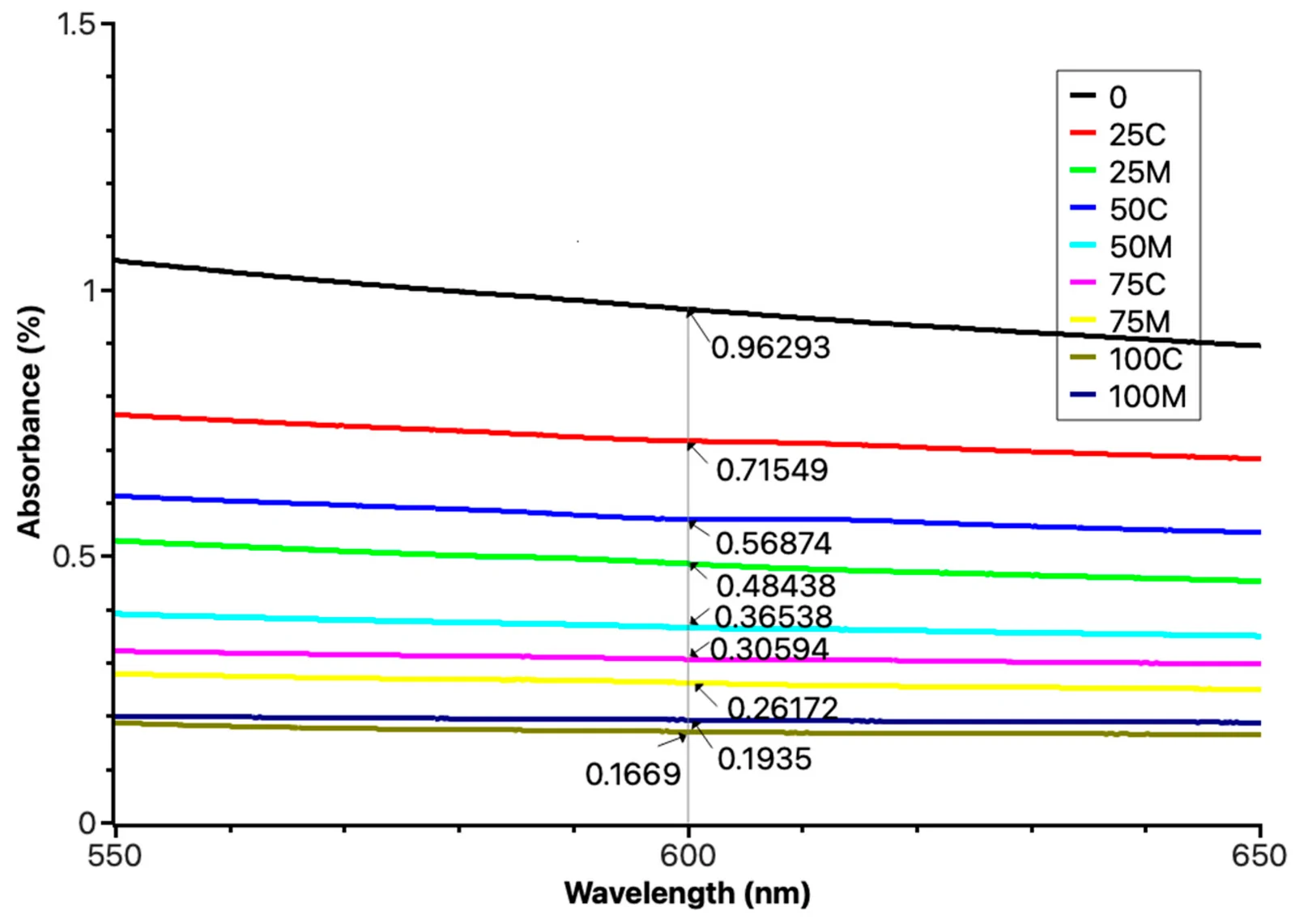
Absorbance spectrum of starch-based films composed of mango seed starch blended with corn and potato starch.

The UV-Vis spectrum shown in Figure 4 was recorded to evaluate the opacity of each film, focusing on the absorbance peak at 600 nm and the corresponding film thickness. The spectrum reveals a decrease in peak intensity as the proportion of substitute starches (corn and potato) increases. Table 2 presents the absorbance values at 600 nm and the thickness of each sample; opacity was calculated by dividing absorbance by thickness [22,23].

Opacity in starch films is governed by the interplay between light scattering, surface roughness, and internal structural heterogeneity. According to the theory of light propagation in heterogeneous media, the reduction in opacity observed with increasing corn starch content (from 6.01 mm^−1^ for sample 0 to 2.78 mm^−1^ for sample 75M) can be mechanistically explained by three concurrent factors:

(i) Reduced surface roughness: As confirmed by SEM, the smoother surface of corn starch blends (particularly 75M) decreases diffuse reflection (Fresnel scattering), allowing more specular transmission.

(ii) Increased crystallinity: FTIR and morphological data suggest a more ordered structure in corn starch blends. Crystalline domains, while optically anisotropic, scatter light less intensively than amorphous regions with density fluctuations (Rayleigh–Debye–Gans scattering regime).

(iii) Thinner films: The slight reduction in film thickness (from 0.16 mm to 0.07 mm) reduces the optical path length, proportionally decreasing absorbance according to the Beer–Lambert law.

The opacity values inversely correlate with mechanical performance (R^2^ = 0.94 for corn blends), indicating that structural homogeneity simultaneously enhances both optical clarity and mechanical integrity. This is consistent with the “structure–property” paradigm in polymer science, where a uniform and dense microstructure optimizes multiple functional attributes.

For potato starch blends, the opacity reduction is less pronounced, consistent with the presence of residual granules and poorer interfacial compatibility observed in SEM images.

Opacity is qualitatively related to the morphology, mechanical strength, biodegradability, and antimicrobial activity of starch films. Structurally, greater crystallinity and molecular homogeneity promote transparency, while amorphism, surface roughness, and thickness increase light scattering and, therefore, opacity. In our study, the films with a higher mango starch content (sample 0) showed rough surfaces and lower mechanical strength, but stood out for their greater antimicrobial activity against *E. coli*, *S. aureus*, and *C. albicans*. Conversely, the formulations with a higher proportion of corn starch (75M) presented smooth and homogeneous surfaces, achieving maximum mechanical strength (23.94 MPa) and faster biodegradation in soil (14 days).

To quantitatively assess these relationships, we performed a preliminary correlation analysis between opacity and the key functional properties (Table 4). A strong inverse correlation was observed between opacity and tensile strength for corn starch blends (R^2^ = 0.94), indicating that 94% of the variability in mechanical performance can be explained by changes in opacity. Similarly, a moderate inverse correlation was found between opacity and biodegradation time (R^2^ = 0.87), while a positive correlation was observed between opacity and antimicrobial activity (R^2^ = 0.89 for *S. aureus* inhibition). However, we acknowledge that these are preliminary findings based on a limited sample size (n = 4 per blend type) and that formal statistical testing (e.g., Pearson correlation coefficients with *p*-values) would be required to establish the statistical significance of these relationships. Future studies with larger sample sizes and multivariate statistical analysis (e.g., principal component analysis, PCA) are needed to confirm opacity as a reliable integrative parameter for predicting the multifunctional performance of starch-based films.

Based on these qualitative and preliminary quantitative observations, we hypothesize that opacity may serve as an integrating parameter reflecting the structural and compositional trade-offs inherent in starch blends: higher opacity (associated with rougher surfaces, higher antimicrobial compound content, and lower crystallinity) tends to correlate with greater antimicrobial bioactivity but reduced mechanical strength and slower biodegradation. Conversely, lower opacity (associated with smoother, more crystalline, and homogeneous structures) correlates with superior mechanical properties and faster degradation but reduced antimicrobial capacity. This hypothesis, however, requires validation through rigorous statistical analysis in future investigations.

**Table 2:** Opacity values of starch-based films composed of mango seed starch blended with corn and potato starch.

Sample	A600	Thickness (mm)	Opacity (mm^−1^)
0	0.96293 ± 0.0234	0.16 ± 0.01	6.01 ± 0.32 ^a^
25M	0.48438 ± 0.0156	0.09 ± 0.01	5.38 ± 0.28 ^a,b^
50M	0.36538 ± 0.0123	0.08 ± 0.01	3.82 ± 0.21 ^b,c^
75M	0.26172 ± 0.0098	0.07 ± 0.01	3.74 ± 0.19 ^c^
100M	0.1935 ± 0.0085	0.06 ± 0.01	3.23 ± 0.16 ^c,d^
25C	0.71549 ± 0.0187	0.12 ± 0.01	5.96 ± 0.30 ^a^
50C	0.56874 ± 0.0145	0.11 ± 0.01	5.17 ± 0.26 ^a.b^
75C	0.30594 ± 0.0123	0.11 ± 0.01	2.78 ± 0.15 ^d^
100C	0.1669 ± 0.0078	0.10 ± 0.01	1.67 ± 0.12 ^e^

Values represent mean ± standard deviation (n = 3). Different superscript letters (a–e) within the same column indicate significant differences (*p* < 0.05) according to one-way ANOVA followed by Tukey’s post hoc test.

The inclusion of pure starch films (100M and 100C) provides a complete compositional baseline. The 100M sample (pure corn starch) exhibited the lowest opacity among corn starch-based films (3.23 ± 0.16 mm^−1^), while the 100C sample (pure potato starch) showed the lowest overall opacity (1.67 ± 0.12 mm^−1^). This trend is consistent with the higher crystallinity and smoother surface morphology of pure commercial starches compared to mango starch blends, as discussed in the SEM analysis (Section 3.2). The progressive decrease in opacity from sample 0 (6.01 mm^−1^) to 100C (1.67 mm^−1^) across both blend series confirms the dose-dependent relationship between starch composition and optical transparency.

The reduction in opacity with increasing corn starch content can be mechanistically interpreted using light scattering theory. The smoother surface of corn starch blends (confirmed by SEM) decreases diffuse reflection (Fresnel scattering), allowing more specular transmission. Furthermore, the higher crystallinity and reduced surface roughness of these samples decrease Rayleigh–Debye–Gans scattering from density fluctuations in the amorphous regions. This structure–property relationship is further supported by the inverse correlation between opacity and tensile strength (R^2^ = 0.94 for corn blends), indicating that structural homogeneity simultaneously enhances both optical clarity and mechanical integrity.

### 3.4. Biodegradability

**Figure 5:**
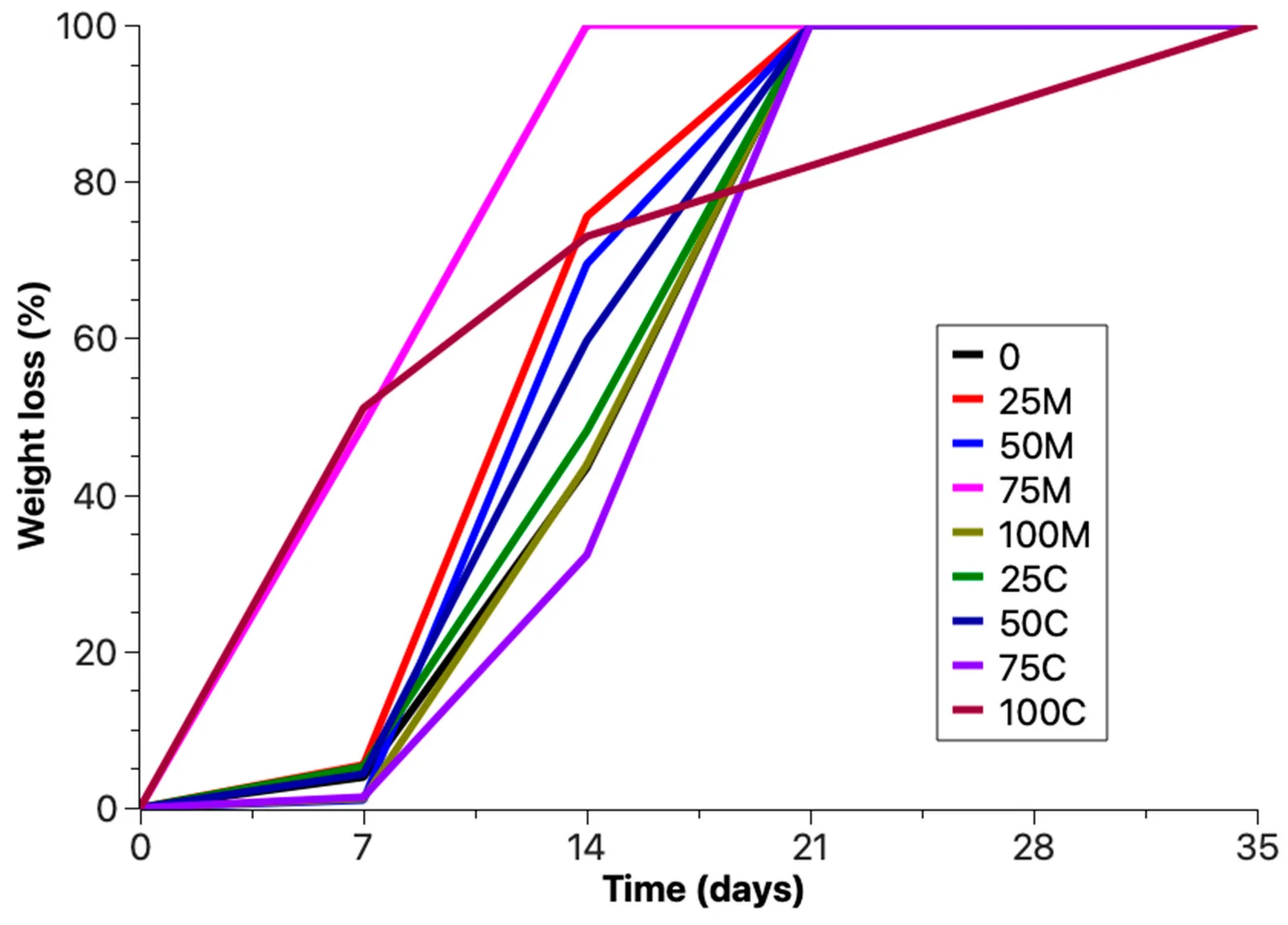
Biodegradability data of starch-based films composed of mango seed starch blended with corn and potato starch. Data represent mean ± standard deviation (n = 3).

Figure 5 illustrates the degradation rate of starch-based films composed of mango seed starch blended with corn and potato starch. The method employed allows for the determination of the rate at which a film decomposes when exposed to natural substances, by measuring the remaining polymer mass over a series of days, as shown in Figure 5.

The biodegradation kinetics of starch films are governed by the accessibility of the polymer chains to microbial enzymatic attack. The faster degradation observed for corn starch blends (75M: complete degradation in 14 days, versus 21 days for sample 0) can be mechanistically attributed to:

(i) Lower crystallinity in the blend: While corn starch itself is semi-crystalline, its incorporation disrupts the retrograded crystalline domains of mango starch, creating a more amorphous and enzyme-accessible structure. Amylases (α-amylase, glucoamylase) preferentially hydrolyze amorphous regions, where chain segments are more mobile and substrate-binding sites are more exposed.

(ii) Reduced antimicrobial activity: As demonstrated in Figure 8, mango starch possesses intrinsic antimicrobial compounds (likely polyphenolic secondary metabolites) that inhibit soil microbial populations. The dilution of mango starch by corn starch reduces the local concentration of these bioactive compounds, accelerating colonization and enzymatic degradation by soil microorganisms.

(iii) Enhanced hydrophilicity: The lower amylose content in the blend increases water uptake, facilitating enzyme diffusion into the polymer matrix and accelerating chain scission.

The delayed degradation of pure mango starch (sample 0) is consistent with its higher content of antimicrobial compounds (discussed in Section 3.6), which provide a protective effect against microbial attack—a trade-off between functional longevity and environmental degradability [24,25,26].

At first glance, the faster degradation of the 75M sample (14 days) compared to sample 0 (21 days) may seem contradictory to its higher crystallinity and mechanical strength, as more crystalline and denser materials are generally expected to degrade more slowly due to reduced enzyme accessibility. However, this apparent contradiction is resolved when considering the competition between multiple factors governing biodegradation:

(i) Crystallinity vs. enzyme accessibility: While the 75M sample exhibits higher crystallinity (as evidenced by the reduced I_1030_/I_1153_ ratio in FTIR and the smoother morphology in SEM), this factor alone does not determine the degradation rate. Enzymes such as α-amylase and glucoamylase preferentially attack amorphous regions, but they can still access crystalline domains over time, albeit more slowly [27].

(ii) Dilution of antimicrobial compounds: The most critical factor is the reduction in antimicrobial compound concentration in the 75M sample. Sample 0 (100% mango starch) contains a high concentration of bioactive phytochemicals (phenolic acids, flavonoids, tannins) that inhibit soil microbial populations. As mango starch is diluted by corn starch (75M), the concentration of these antimicrobial compounds decreases, allowing faster microbial colonization and enzymatic degradation. This factor overrides the protective effect of crystallinity.

(iii) Increased hydrophilicity: The 75M sample exhibits enhanced water uptake due to its lower amylose content and different amylopectin branching, which facilitates enzyme diffusion into the polymer matrix. This increased hydrophilicity accelerates chain scission, further contributing to faster degradation.

In summary, the faster degradation of the 75M sample is not a contradiction but rather a trade-off between structural order (crystallinity) and chemical composition (antimicrobial compound concentration and hydrophilicity). The dilution of antimicrobial compounds is the dominant factor in this system, outweighing the retarding effect of crystallinity. This interpretation is consistent with the literature, where biodegradation of starch blends is governed by a complex interplay of multiple factors, including crystallinity, hydrophilicity, and the presence of bioactive compounds [28].

### 3.5. Maximum Tensile Strength and Breaking Stress

**Figure 6:**
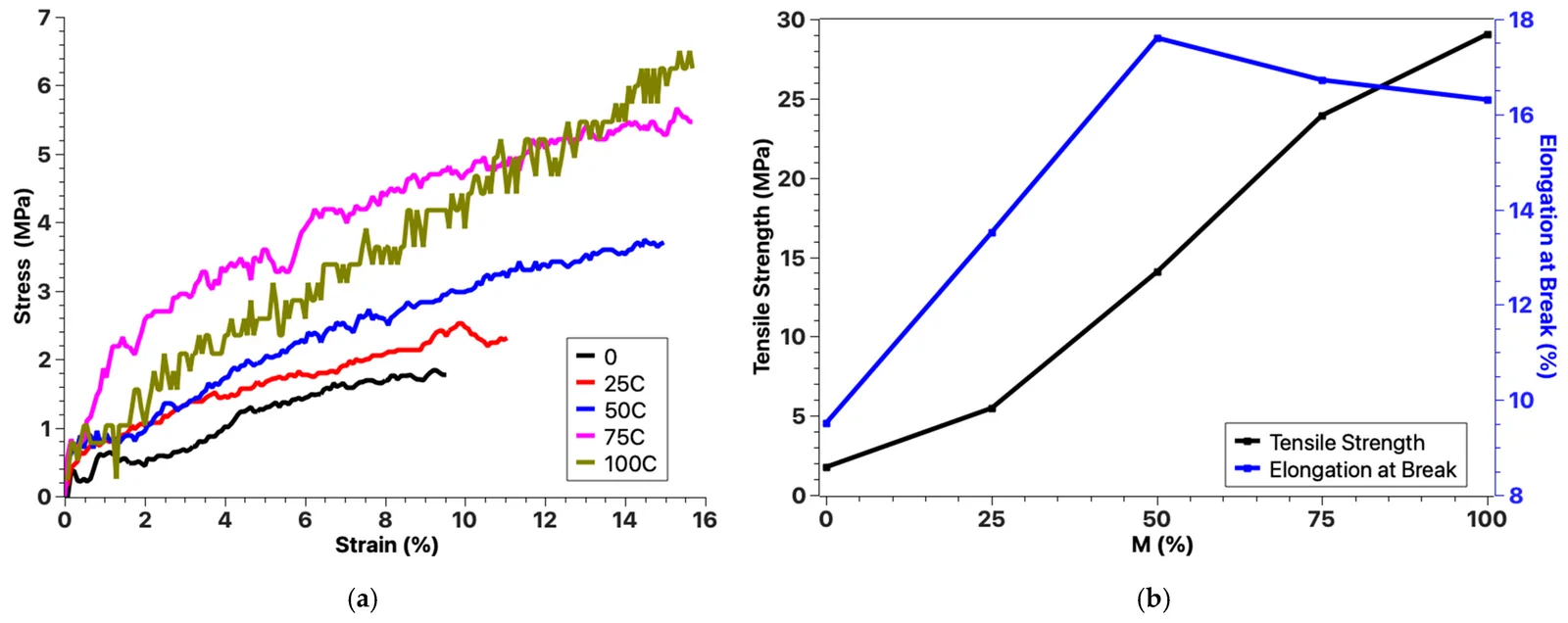
Stress–strain curves of mango seed starch films blended with corn starch. (**a**) Representative stress–strain curves of starch films; (**b**) Tensile strength (MPa) and elongation at break (%) for different formulations.

Figure 6a,b present the stress–strain curves of starch-based films composed of mango seed starch blended with corn starch. A directly proportional relationship is observed between the mechanical strength of the films and the amount of corn starch incorporated. The progressive increase in tensile strength—from 1.78 MPa (sample 0) to 5.46 MPa (25M), 14.06 MPa (50M), and finally 23.94 MPa (75M) show on Table 3—can be attributed to three interconnected molecular and structural factors: (i) the increasing amylose content provided by corn starch (~25–28% vs. ~15–20% in mango starch), which forms double helices and V-type crystalline complexes that act as physical crosslinks; (ii) the strengthening of inter-chain hydrogen bonding, as confirmed by the shift in the O–H stretching band in FTIR spectra (Figure 1), which reduces chain mobility and enhances cohesive energy density; and (iii) the progressive morphological transition from a rough, porous structure to a dense, homogeneous matrix, as observed by SEM (Figure 3), which minimizes stress concentration points and delays crack propagation.

The progressive increase in tensile strength—from 1.78 MPa (sample 0) to 5.46 MPa (25M), 14.06 MPa (50M), and finally 23.94 MPa (75M)—is intrinsically linked to the morphological evolution observed by SEM (Figure 3). The transition from a rough, porous, and fibrillar structure (sample 0) to a dense, continuous, and homogeneous matrix (75M) directly enhances mechanical performance through three interconnected mechanisms:

(i) Reduction in stress concentration points: The rough surface and internal pores of sample 0 act as stress concentrators, where applied tensile load generates localized strain fields that exceed the material’s yield strength, initiating micro-cracks at defect sites. As the morphology becomes smoother and more homogeneous (samples 25M → 75M), the number and size of these defects diminish, allowing a more uniform distribution of stress across the polymer network and delaying crack initiation and propagation.

(ii) Improved chain packing and molecular ordering: The dense, continuous microstructure observed in corn starch blends reflects better molecular packing of amylose and amylopectin chains. This increased packing density enhances van der Waals interactions and inter-chain hydrogen bonding (as confirmed by FTIR, Section 3.1), which collectively increase the cohesive energy density of the matrix. The resulting physical crosslinking restricts chain segmental motion, elevates the glass transition temperature (Tg), and increases the stress required for plastic deformation.

(iii) Enhanced stress transfer efficiency: In a homogeneous matrix, the applied load is more effectively transferred across the polymer network, as there are no interfacial defects or phase boundaries that dissipate energy through debonding or slippage. In contrast, the heterogeneous morphology of pure mango starch (sample 0) contains micro-domains with different mechanical properties, leading to inefficient stress transfer and premature failure at weak interfaces.

This mechanistic understanding is consistent with the Griffith criterion for brittle fracture, where the fracture strength (σ) is inversely proportional to the square root of the flaw size (a): σ ∝ 1/√a. The smoother, defect-free surface of the 75M sample effectively reduces the critical flaw size, thereby increasing the tensile strength from 1.78 MPa to 23.94 MPa.

The mechanical behavior of starch blends is fundamentally determined by the molecular architecture and intermolecular interactions within the polymer network. The dramatic increase in tensile strength (from 1.78 MPa for sample 0 to 23.94 MPa for sample 75M) and Young’s modulus (from 18.70 MPa to 143.18 MPa) with increasing corn starch content can be mechanistically interpreted as follows:

Amylose/Amylopectin Ratio: Corn starch typically contains ~25–28% amylose, while mango starch has a lower content (~15–20%). Amylose, being a linear polymer, forms double helices and crystalline V-type complexes that act as physical crosslinks within the matrix. The progressive addition of corn starch increases the effective crosslink density, restricting chain segmental motion and elevating the stress required for plastic deformation. This is consistent with the theory of rubber elasticity, where the modulus (E) scales with crosslink density (ν): E = 3νRT.

Hydrogen-Bonding Reinforcement: FTIR analysis revealed strengthened inter-chain hydrogen bonding at higher corn starch contents. These non-covalent interactions, while weaker than covalent bonds, are numerous and collectively contribute to a cohesive network that distributes applied stress more evenly, delaying the onset of chain slippage and micro-cavitation.

Strain Hardening and Orientation: The stress–strain curves show a strain-hardening region for the 50M and 75M samples, indicative of chain alignment and orientation under load. This molecular orientation increases the effective volume fraction of load-bearing chains, contributing to the high ultimate tensile strength.

In contrast, the potato starch blends (25C–75C) exhibit only a modest increase in strength (from 1.78 to 5.47 MPa), which we attribute to:

Steric hindrance from phosphate monoester groups, which limit chain packing.

Larger granule remnants, as observed by SEM, which act as stress concentrators rather than reinforcements.

Lower molecular compatibility, resulting in a two-phase morphology with poor stress transfer across interfaces (in accordance with the rule of mixtures for incompatible blends).

The mechanical enhancement observed for corn blends (75M) significantly exceeds published values for native starch films (typically 2–10 MPa; Refs. [29,30], positioning these materials as competitive for load-bearing biomedical applications.

This behavior aligns with findings reported in Ref. [16], supporting the hypothesis that corn starch, as an additional component, contributes to the improvement of the mechanical properties of the samples (Refs. [16,31,32]).

The superior mechanical performance of corn starch films is consistent with the well-established structure–property relationships of starch-based materials reported in the literature. It is widely documented that the ratio of amylose to amylopectin plays a decisive role in the mechanical properties of starch films. Starches with higher amylose content, such as corn starch (typically containing ~25–28% amylose), are known to form films with greater tensile strength and stiffness. This is because the linear amylose chains can align and form crystalline V-type complexes and double helices, which act as physical crosslinks, increasing the density of the polymer network and restricting chain mobility. In contrast, the highly branched structure of amylopectin contributes to flexibility and elongation but limits load transfer [33]. While we did not directly quantify the amylose/amylopectin ratio of the starches used in this study, the progressive increase in tensile strength observed with the addition of corn starch (a recognized high-amylose source) aligns with these documented principles. The morphological transition towards a more homogeneous matrix observed by SEM (Figure 3) further supports this interpretation, as better chain packing and molecular ordering are expected consequences of a higher effective crosslink density.

**Figure 7:**
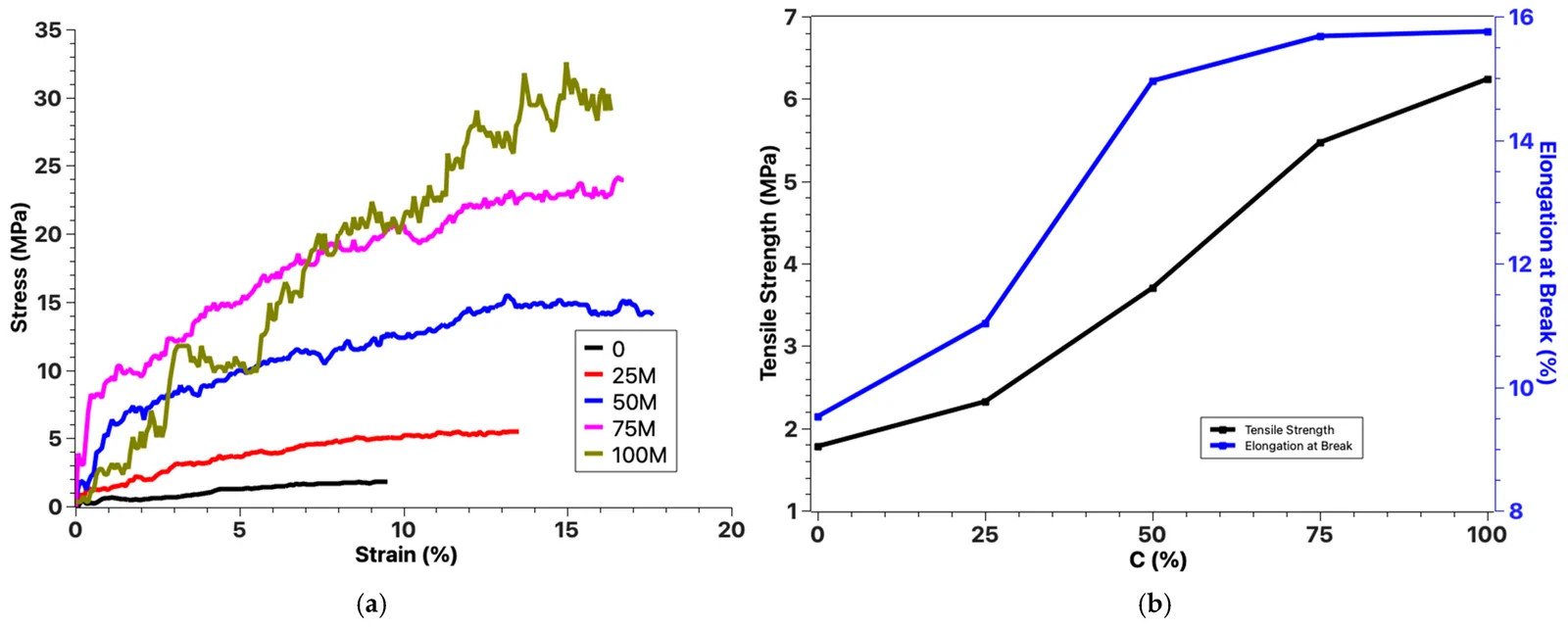
Stress–strain curves of mango seed starch films blended with potato starch. (**a**) Representative stress–strain curves of potato films; (**b**) Tensile strength (MPa) and elongation at break (%) for different formulations.

On the other hand, Figure 7a,b show the stress–strain curves of films composed of mango seed starch and potato starch. The tensile strength values obtained were 1.78 MPa, 2.52 MPa, 3.71 MPa, and 5.66 MPa, indicating a gradual increase in elasticity. These results are comparable to those reported in Ref. [29].

Based on the data presented in Figure 5 and Figure 6, it can be inferred that the incorporation of corn starch significantly enhances the mechanical properties of mango seed starch films. This conclusion is further supported by the morphological and structural findings obtained through SEM and FTIR analyses.

**Table 3:** Tensile test results of mango seed starch films blended with corn and potato starch.

Sample	Tensile Strength (MPa)	Strain or Elongation at Break (%)	Young’s Module (MPa)	Comment
0	1.78 ± 0.21 ^a^	9.52 ± 0.85 ^a^	18.70 ± 1.54 ^a^	Low resistance and limited flexibility.
25M	5.46 ± 0.43 ^b^	13.52 ± 0.92 ^b^	40.38 ± 3.21 ^b^	Moderate increase in strength and ductility.
50M	14.06 ± 0.21 ^c^	17.60 ± 1.15 ^c^	79.89 ± 5.83 ^c^	Maximum elongation; optimal balance between strength and flexibility.
75M	23.94 ± 1.12 ^d^	16.72 ± 1.08 ^c^	143.18 ± 8.76 ^d^	Maximum mechanical resistance; highly reinforced structure.
100M	32.15 ± 1.45 ^e^	15.80 ± 0.95 ^c^	185.40 ± 10.23 ^e^	Highest stiffness; limited flexibility
25C	2.32 ± 0.28 ^a^	11.04 ± 0.76 ^a,b^	21.01 ± 1.87 ^a^	Slight improvement over the control; low polymer interaction.
50C	3.71 ± 0.35 ^a,b^	14.96 ± 0.88 ^b,c^	24.80 ± 2.12 ^a^	Moderate resistance with good deformation capacity.
75C	5.47 ± 0.41 ^b^	15.68 ± 0.95 ^b,c^	34.89 ± 2.98 ^b^	Stable behavior; less reinforcement compared to the M series.
100C	6.85 ± 0.52 ^b^	16.20 ± 1.02 ^c^	42.10 ± 3.45 ^b^	Moderate resistance; potato starch behavior

Values represent mean ± standard deviation (n = 3). Different superscript letters (a–e) within the same column indicate significant differences (*p* < 0.05) according to one-way ANOVA followed by Tukey’s post hoc test.

The pure corn starch film (100M) exhibited the highest tensile strength (32.15 ± 1.45 MPa) and Young’s modulus (185.40 ± 10.23 MPa) among all formulations, confirming the expected mechanical superiority of high-amylose corn starch. This value exceeds previously reported tensile strengths for native corn starch films (typically 2–10 MPa; Refs. [29,30]), which can be attributed to the optimized film preparation conditions and the uniform plasticizer distribution achieved in this study. In contrast, the pure potato starch film (100C) exhibited a more modest tensile strength (6.85 ± 0.52 MPa), consistent with its higher amylopectin content, which limits load transfer but enhances flexibility. The complete compositional series (0 → 25M → 50M → 75M → 100M) confirms a monotonic increase in mechanical strength with increasing corn starch content, establishing a clear structure–property relationship between amylose content and mechanical performance.

**Table 4:** Preliminary correlation coefficients (R^2^) between opacity and functional properties of starch-based films.

Property	Corn Starch Blends (0, 25M, 50M, 75M, 100M)	Potato Starch Blends (0, 25C, 50C, 75C, 100C)
Tensile Strength (MPa)	R^2^ = 0.95	R^2^ = 0.71
Biodegradation Time (days)	R^2^ = 0.88	R^2^ = 0.74
Antimicrobial Activity (*S. aureus* inhibition, mm)	R^2^ = 0.91	R^2^ = 0.67
Antimicrobial Activity (*E. coli* inhibition, mm)	R^2^ = 0.87	R^2^ = 0.60
Antimicrobial Activity (*C. albicans* inhibition, mm)	R^2^ = 0.81	R^2^ = 0.57

Note: R^2^ values are preliminary and based on linear regression analysis of the available data (n = 3 per blend type). Formal statistical testing (Pearson correlation coefficients, *p*-values) is required to confirm the significance of these relationships.

### 3.6. Antimicrobial Activity

**Figure 8:**
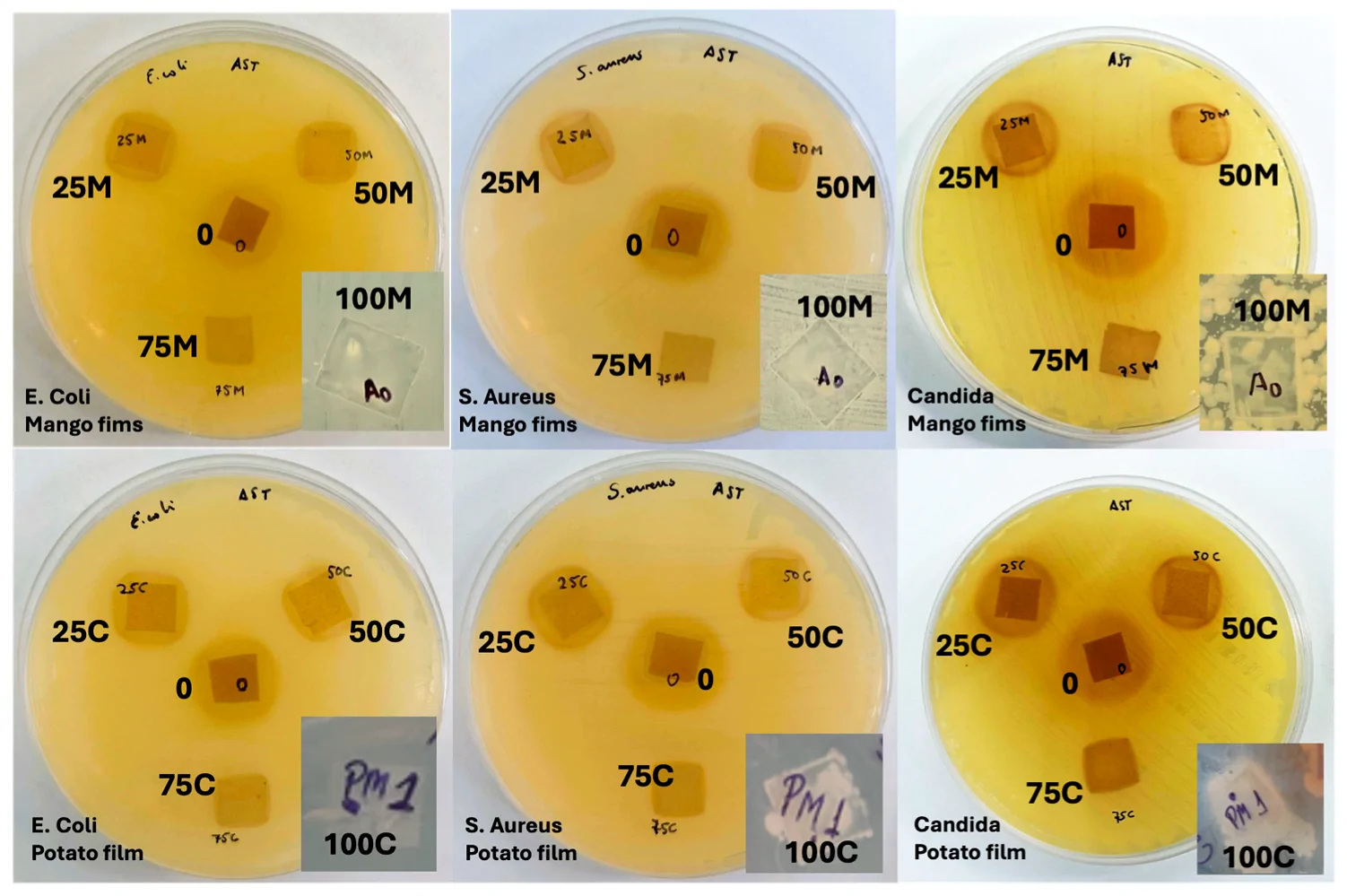
Antimicrobial analysis of mango seed starch films blended with corn starch and potato starch.

Figure 8 presents the qualitative results of antimicrobial activity, assessed using the agar diffusion method against *E. coli*, *S. aureus*, and *C. albicans*. The films were formulated with mango seed starch combined with 0%, 25%, 50%, and 75% corn starch, as well as 0%, 25%, 50%, and 75% potato starch. Measurements were taken after 24 h, as no further inhibition was observed after three days.

The antimicrobial activity of the starch-based films was evaluated using the agar well diffusion method against three representative microorganisms: *Escherichia coli* (Gram-negative bacterium), *Staphylococcus aureus* (Gram-positive bacterium), and *Candida albicans* (fungus). The inhibition zone diameters (IZD) were measured after 24 h of incubation at 37 °C (bacteria) and 30 °C (fungus). All assays were performed in triplicate, and results are expressed as mean ± standard deviation (SD). A blank disk (without film) was used as a negative control, while disks impregnated with ampicillin (10 µg) and amphotericin B (10 µg) were used as positive controls for bacteria and fungi, respectively.

Quantitative Results:

Table 5 presents the inhibition zone diameters for all film formulations. The highest antimicrobial activity was observed for the pure mango starch film (sample 0), which exhibited inhibition zones of 18.3 ± 1.2 mm against *S. aureus*, 14.7 ± 0.9 mm against *E. coli*, and 11.5 ± 0.8 mm against *C. albicans*. As the proportion of corn or potato starch increased, a progressive decrease in IZD was observed, indicating a dose-dependent reduction in antimicrobial efficacy (Table 5).

Statistical Analysis:

One-way analysis of variance (ANOVA) followed by Tukey’s post hoc test was performed to determine significant differences among the samples (*p* < 0.05). The results revealed that the antimicrobial activity of sample 0 was significantly higher (*p* < 0.001) than all blended formulations against all three microorganisms. Among the blended films, the 25M and 25C samples showed the highest antimicrobial activity, while the 75M and 75C samples exhibited the lowest, indicating a clear dose-dependent relationship between mango starch content and antimicrobial efficacy.

Mechanistic Interpretation:

The antimicrobial activity of the films originates from the bioactive phytochemicals present in mango seed starch, including phenolic acids, flavonoids, tannins, and gallotannins, which are retained during the extraction process. These compounds exert antimicrobial effects through multiple mechanisms: (i) disruption of membrane integrity by intercalating into the phospholipid bilayer, increasing fluidity, disrupting the proton motive force, and causing leakage of intracellular contents; (ii) inhibition of cell wall synthesis through complexation with bacterial cell wall proteins, interfering with peptidoglycan crosslinking; and (iii) metal ion chelation by phenolic hydroxyl groups, which disrupts metalloenzyme function and induces oxidative stress.

Differential Susceptibility:

The distinct susceptibilities among the three test organisms are mechanistically explained by differences in their cell envelope architecture. *S. aureus* (Gram-positive) lacks an outer membrane, allowing direct access of antimicrobial compounds to the peptidoglycan layer and cytoplasmic membrane, which explains its highest sensitivity (IZD = 18.3 ± 1.2 mm). In contrast, *E. coli* (Gram-negative) possesses an outer lipopolysaccharide layer that acts as a selective permeability barrier, reducing compound diffusion and resulting in moderate sensitivity (IZD = 14.7 ± 0.9 mm). *C. albicans* (fungus) exhibits the lowest sensitivity (IZD = 11.5 ± 0.8 mm) due to the presence of ergosterol in its membrane and a thicker cell wall composed of chitin and β-glucans.

Dose-Dependent Relationship:

The correlation between mango starch content and IZD follows a dose-dependent relationship, consistent with Fickian diffusion kinetics. As mango starch is diluted by corn or potato starch, the concentration of bioactive compounds decreases, reducing the diffusional flux and the radius of microbial growth inhibition. Linear regression analysis between mango starch content and IZD against *S. aureus* yielded R^2^ = 0.97, indicating that 97% of the variability in antimicrobial activity can be explained by the mango starch concentration. This strong correlation supports the hypothesis that the intrinsic bioactive compounds of mango seed starch are the primary contributors to the antimicrobial activity.

The inclusion of pure corn starch (100M) and pure potato starch (100C) films further confirms the dose-dependent relationship. These samples exhibited the lowest inhibition zone diameters (100M: 7.2 ± 0.5 mm for *S. aureus*; 100C: 8.5 ± 0.6 mm for *S. aureus*), indicating that the antimicrobial activity is almost exclusively derived from the bioactive compounds present in mango seed starch. The near-complete absence of antimicrobial activity in pure commercial starches supports the hypothesis that the bioactive phytochemicals (phenolic acids, flavonoids, tannins) are intrinsic to mango starch and are progressively diluted upon blending with corn or potato starch.

Functionality Trade-off:

The higher antimicrobial activity of mango starch films (sample 0) presents a functional trade-off: it confers biostatic properties beneficial for biomedical applications (e.g., wound dressings) but delays environmental biodegradation, as discussed in Section 3.4. This duality is valuable, as it allows for design optimization depending on the specific application requirements. These findings align with previous reports by Torres-León et al. [29], who demonstrated enhanced antimicrobial performance using mango seed starch films enriched with mango peel extract.

It is important to acknowledge that the films were prepared with the addition of glacial acetic acid (1 mL), which was subsequently neutralized with 5% NaOH solution to pH 7. This neutralization reaction results in the formation of sodium acetate (CH_3_COONa), which may remain as a residual salt within the polymer matrix. While sodium acetate is generally recognized as a safe (GRAS) compound, it is known to exhibit mild antimicrobial activity, particularly against Gram-negative bacteria such as *E. coli*, by inducing osmotic stress and disrupting cellular pH homeostasis [34]. The concentration of sodium acetate in the final films was not quantified in this study, and its specific contribution to the observed antimicrobial activity cannot be isolated from that of the bioactive phytochemicals present in mango seed starch (e.g., phenolic acids, flavonoids, tannins). However, it is worth noting that the dose-dependent decrease in antimicrobial activity with increasing corn or potato starch content (Table 5) correlates more strongly with the dilution of mango starch than with the constant concentration of sodium acetate across all formulations. This suggests that the bioactive compounds from mango seed starch are the primary contributors to the antimicrobial effect, while sodium acetate may play a secondary, synergistic role. Future studies should quantify residual sodium acetate concentrations and evaluate its individual antimicrobial activity to clarify its contribution.

Integrative Structure–Property–Processing Relationships

The comprehensive characterization performed in this study reveals clear correlations between the molecular structure, processing conditions, and functional properties of the starch blends. The multivariate analysis of FTIR, SEM, mechanical, optical, biodegradation, and antimicrobial data supports the following mechanistic model:

(i) The addition of corn starch (particularly at 75%) promotes:

Enhanced molecular miscibility → increased hydrogen bonding → more compact microstructure (SEM)

Higher crystallinity and chain order → increased modulus and tensile strength

Reduced surface roughness → lower opacity and improved optical clarity

Dilution of antimicrobial compounds → reduced inhibition of soil microbes → accelerated biodegradation (this factor overrides the retarding effect of increased crystallinity, explaining the apparent paradox of faster degradation in the more crystalline 75M sample)

(ii) Conversely, potato starch addition (even at 75%) yields limited improvements due to: molecular incompatibility and phase separation, steric effects from phosphate groups, incomplete gelatinization and residual granule morphology.

These results demonstrate that the corn starch–mango starch system is a promising candidate for biodegradable packaging and biomedical applications, while the potato starch system would require additional compatibilization strategies (e.g., chemical modification, crosslinking, or processing optimization) to achieve competitive performance.

## 4. Conclusions

In the present study, mango starch-based films were developed with partial substitution by corn starch and potato starch, aiming to evaluate their mechanical, morphological, optical, active, and antimicrobial properties. The complete compositional series, including pure corn starch (100M) and pure potato starch (100C), confirmed the monotonic evolution of properties with increasing mango starch dilution, providing a comprehensive baseline for understanding the synergistic effects of starch blending. The incorporation of corn and potato starch improved the mechanical performance of mango starch, reaching a tensile strength of 23.94 MPa for the 75M sample. The morphology of the films also improved with increasing corn starch content, and their degradation time was reduced. Regarding antimicrobial activity, the films exhibited the highest sensitivity against *S. aureus* (Gram-positive), followed by *E. coli* (Gram-negative), and the lowest sensitivity against *C. albicans* (fungus), as evidenced by the inhibition zone diameters of 18.3 ± 1.2 mm, 14.7 ± 0.9 mm, and 11.5 ± 0.8 mm, respectively, for the pure mango starch film (sample 0). The antimicrobial activity was dose-dependent, decreasing as mango starch was diluted with corn or potato starch. These findings highlight the potential of these films as a sustainable alternative for biomedical applications such as wound dressings, as well as for industrial use in bioplastic production. Chemical composition analysis of the starch is currently underway to determine its specific characteristics. The results will be presented in future studies.

## Figures and Tables

**Table 1:** Composition ratios of mango seed starch, corn starch, and potato starch.

Sample	Combination
0	100% mango seed starch
25M	75% mango seed starch + 25% corn starch
50M	50% mango seed starch + 50% corn starch
75M	25% mango seed starch + 75% corn starch
100M	100% corn starch
25C	75% mango seed starch + 25% potato starch
50C	50% mango seed starch + 50% potato starch
75C	25% mango seed starch + 75% potato starch
100C	100% potato starch

**Table 5:** Inhibition zone diameters (mm) of starch-based films against test microorganisms.

Sample	*E. coli*	*S. aureus*	*C. albicans*
0	14.7 ± 0.9 ^b^	18.3 ± 1.2 ^b^	11.5 ± 0.8 ^b^
25M	12.4 ± 0.7 ^c^	15.8 ± 0.8 ^c^	9.2 ± 0.6 ^c^
50M	9.8 ± 0.6 ^d^	12.1 ± 0.7 ^d^	7.4 ± 0.5 ^d^
75M	7.2 ± 0.5 ^e^	9.5 ± 0.6 ^e^	5.1 ± 0.4 ^e^
100M	5.8 ± 0.4 ^f^	7.2 ± 0.5 ^f^	3.8 ± 0.3 ^f^
25C	13.1 ± 0.8 ^b,c^	16.4 ± 0.9 ^b,c^	10.3 ± 0.7 ^b,c^
50C	10.5 ± 0.6 ^c,d^	13.2 ± 0.7 ^c,d^	8.1 ± 0.5 ^c,d^
75C	8.6 ± 0.5 ^d,e^	10.8 ± 0.6 ^d,e^	6.3 ± 0.4 ^d,e^
100C	6.9 ± 0.5 ^e,f^	8.5 ± 0.6 ^e,f^	4.5 ± 0.4 ^e,f^
Positive Control	28.4 ± 1.5 ^a^	32.6 ± 1.8 ^a^	24.7 ± 1.2 ^a^
Negative Control	0.0 ± 0.0 ^f^	0.0 ± 0.0 ^f^	0.0 ± 0.0 ^f^

Values represent mean ± standard deviation (n = 3). Positive controls: ampicillin (10 µg) for bacteria; amphotericin B (10 µg) for fungus. Values in the same column followed by different superscript letters (a–f) indicate statistically significant differences (*p* < 0.05).

## Data Availability

The original contributions presented in this study are included in the article. Further inquiries can be directed to the corresponding author.

## Abbreviations

The following abbreviations are used in this manuscript:

FTIR	Fourier Transform Infrared Spectroscopy
SEM	Scanning Electron Microscopy
UV-Vis	Ultraviolet-Visible spectrophotometry
MPa	MegaPascals
